# Complete mitochondrial genome of the Caribbean reef shark, *Carcharhinus perezi* (Carcharhinformes: Carcharhinidae)

**DOI:** 10.1080/23802359.2021.1964394

**Published:** 2021-08-19

**Authors:** Austin J. Gallagher, Oliver N. Shipley, Bo Reese, Vijender Singh

**Affiliations:** a Beneath the Waves, Herndon, VA, USA; b Centre for Ecology and Conservation, College of Life and Environmental Sciences, University of Exeter Cornwall Campus, Penryn, Cornwall, UK; c Biology Department, University of New Mexico, Albuquerque, NM, USA; d Center for Genome Innovation, Institute for Systems Genomics, University of Connecticut, Storrs, CT, USA; e Institute for Systems Genomics, Computational Biology Core, University of Connecticut, Storrs, CT, USA

**Keywords:** Shark, Bahamas, reef shark, *Carcharhinus perezi*, mitochondrial genome

## Abstract

The Caribbean reef shark (*Carcharhinus perezi;* Poey, 1876) is a medium to large-bodied coastal and reef-associated predator found throughout the subtropical and tropical waters of the Atlantic Ocean and Caribbean Sea, although its populations are increasingly threatened by overfishing. We describe the first mitochondrial genome sequence for this species, using Illumina MiSeq sequencing of an individual from The Bahamas. We report the mitogenome sequence of the Caribbean reef shark to be 16,709 bp and composed two rRNA genes, 22 tRNA genes, 13 protein-coding genes, 2 non-coding regions; the D-loop control region and the origin of light-strand replication. We discuss the implications of this new information on future monitoring efforts and conservation measures such as marine protected areas, and urge for greater application of mitochondrial studies of sharks in the Atlantic Ocean.

The Caribbean reef shark *Carcharhinus perezi* (Poey, 1876) is a medium to large-bodied marine predator, commonly associated with reefs throughout its distribution in the subtropical Atlantic Ocean. They are ecologically significant consumers and drive food-web linkages on coral reefs (Heupel et al. 2019), and they are economically valuable to the scuba diving industry (Gallagher and Hammerschlag 2011, Haas et al. 2017) in nations where they occur. Caribbean reef sharks are currently listed as ‘Endangered’ by the IUCN Red List (Carlson et al. 2021), although their absence from many nations where they should be found suggests historical or ongoing overfishing and contemporary population declines (MacNeil et al. 2020). As a result, establishing baseline assessments of Caribbean reef shark populations is needed to inform future spatial conservation measures throughout their range (Dwyer et al. 2020; Gallagher et al. 2020). This species is surprisingly understudied; few genetic studies have been conducted (Bernard et al. 2017), and its mitogenome remains undescribed.

We describe the complete mitochondrial genome of the Caribbean reef shark. A 2 mm muscle sample was taken from a live, mature female individual (177 cm total length, identification tag: 39093) sampled from the waters off Great Exuma, The Commonwealth of The Bahamas (23.726984 N, −76.033982 W) on 20 February 2019 (the shark was released alive). The sample was deposited and stored frozen at the Center for Genome Innovation (CGI) at the University of Connecticut (USA) under accession BTW_202039093 (Bo Reese, bo.reese@uconn.edu).

Genomic DNA was extracted using the DNeasy Blood & Tissue Kit (Qiagen, Hilden, Germany) following manufacturer protocol. Genomic DNA (deposited and stored at the CGI) was quantified, with purity ratios determined using the NanoDrop 2000 spectrophotometer (Thermo Fisher Scientific, Waltham, MA, USA). To further assess DNA quality, genomic DNA was analyzed on the Agilent TapeStation 4200 (Agilent Technologies, Santa Clara, CA, USA) using the Genomic DNA assay. Whole genome shotgun libraries were made using the Illumina Nextera XT library preparation kit following manufacturer protocol (Illumina, San Diego, CA). Libraries were validated for length and adapter dimer removal using the Agilent TapeStation 4200 D1000 High Sensitivity assay (Agilent Technologies, Santa Clara, CA, USA) then quantified and normalized using the dsDNA High Sensitivity Assay for Qubit 3.0 (Life Technologies, Carlsbad, CA, USA). Libraries were prepared for sequencing on the Illumina MiSeq using version 2 chemistry for paired end 250 bp reads.

The mitochondrial genome of the Caribbean reef shark is similar to most animal mitogenomes and is 16,709 bp long containing 13 protein coding, 22 tRNA, and 2 rRNA genes, and 1 non-coding control region (Supplemental Information, Table 1). The nucleotide base composition of the entire genome is 31.56% A, 29.89% T, 25.52% C, and 13.01% G, which generated a 61.45% A.T rich feature. The tRNA genes range from 67-75 bp in size, and only tRNASer(GCT) could not fold into a distinctive cloverleaf secondary structure estimating by the tRNAscan-SE v1.21 (Schattner et al., 2005). Among the mitochondrial protein-coding genes, the ND5 is the longest, while the ATP8 is the shortest. The usage of the start codon was mainly ATG except for the COI gene using the GTG; the usage of the stop one was TAA, AGA or T–.

A MUSCLE alignment was performed in Geneious Prime (2021.0.3, https://www.geneious.com/) with the mitochondrial DNA sequence from *Carcharhinus falciformis*, *Carcharhinus leucas*, *Carcharhinus amblyrhynchos*, *Carcharhinus plumbeus*, *Carcharhinus obscurus*, *Carcharhinus melanopterus*, *Carcharhinus albimarginatus*, *Galeocerdo cuvier*, *Carcharhinus acronotus*, *Carcharhinus macloti*, *Carcharhinus sorrah*, *Carcharhinus longimanus*, *Sphyrna mokarran*, *Sphyrna lewini* and *Sphyrna zygaena* species using *Alopias pelagicus* mitochondrion complete genome as the outgroup. A Bayesian tree (Figure 1) was created with default parameters in MrBayes 3.2.6 (Huelsenbeck and Ronquist 2001) and the substitution model (HKY85) determined by the Bayesian Inf.

**Figure 1.**
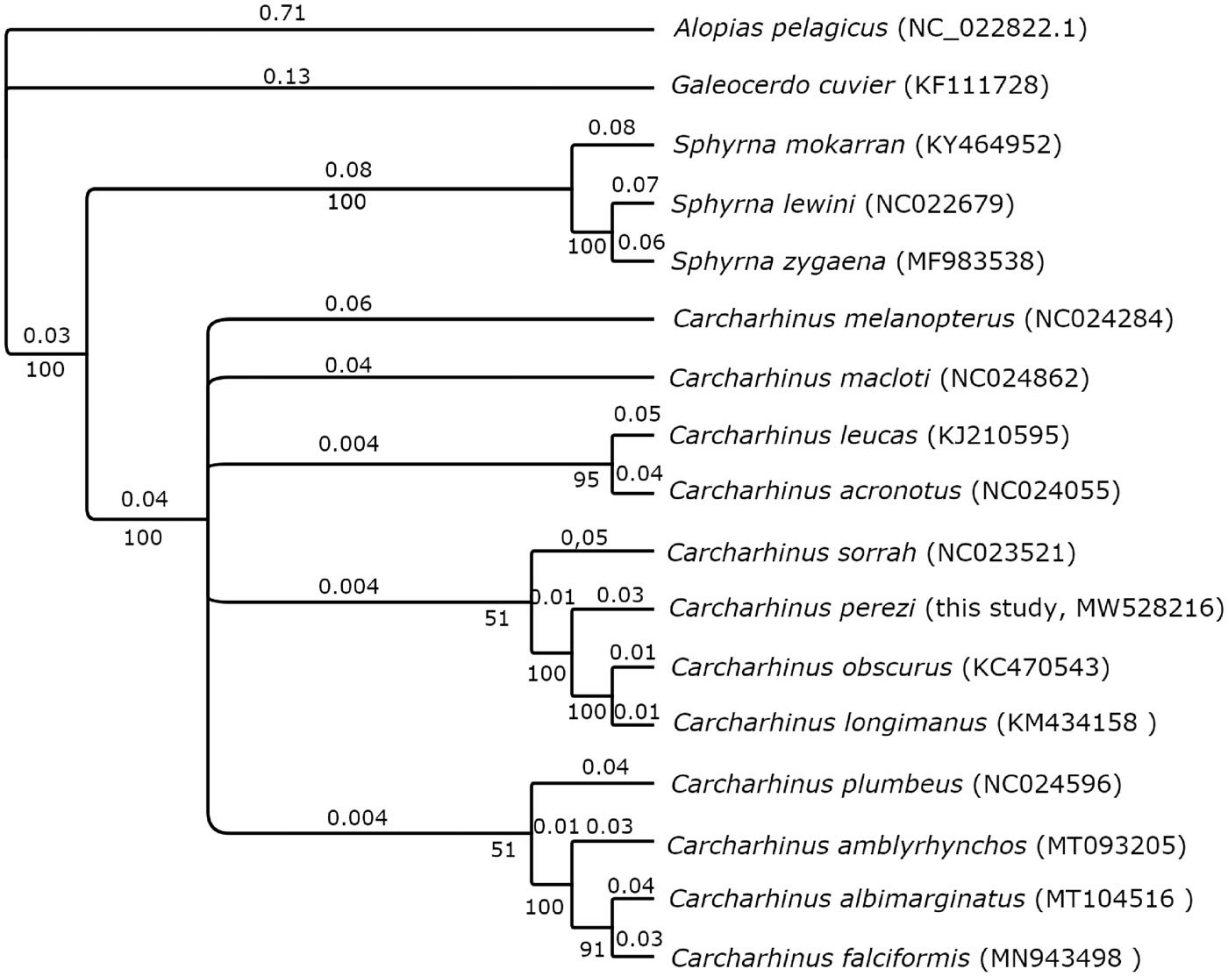
Bayesian inference tree of the phylogenetic relationships among 16 shark species within the Order Carcharhiniformes. Outgroup species *Alopias pelagicus* is from Order Lamniformes. Numbers on branches indicate posterior probabilities in percentage and branch length is proportional to the amount of genetic change (nucleotide substitutions per site). The new sequence for the Caribbean reef shark *Carcharhinus perezi* is included. GenBank accession numbers for each sequence are in parentheses.

The mitogenome of the Caribbean reef shark is similar in length and composition to other sequenced reef shark species (Dunn et al. 2020, Johri et al. 2020). Our study also represents the first shark mitogenome to be sequenced from The Bahamas, a global hotspot for shark biodiversity, and represents one of the few published shark mitogenomes from the Atlantic Ocean. Caribbean reef sharks are completely protected within The Bahamas shark sanctuary, and recent research has demonstrated high residency across various life stages for the species (Shipley et al. 2017, Gallagher et al. 2021), suggesting that this type of large marine protected area (MPA) is likely to be effective over long-term periods. This shark mitogenome will permit more detailed population genetic studies of Caribbean reef shark connectivity, should support future studies evaluating the conservation genetics of reef-associated sharks in the Atlantic, and may assist with the development of innovative methodologies (e.g. eDNA) to monitor their populations as research is expanded within the Greater Caribbean region.

## Data Availability

The genome sequence data that support the findings of this study are openly available in GenBank of NCBI at [https://www.ncbi.nlm.nih.gov] (https://www.ncbi.nlm.nih.gov/) under the accession no. MW528216 (BankIt2420492). The associated data files can be found under BioProject and Bio-Sample numbers are PRJNA731863 and SAMN19298797 respectively.
